# Comparison of two culture techniques used to detect environmental contamination with *Salmonella enterica* in a large-animal hospital

**DOI:** 10.4102/jsava.v86i1.1292

**Published:** 2015-08-13

**Authors:** Catriona H. Lyle, Cornelius H. Annandale, Johan Gouws, Paul S. Morley

**Affiliations:** 1 Department of Companion Animal Clinical Studies, University of Pretoria, South Africa; 2 Department of Production Animal Studies, University of Pretoria, South Africa; 3 Department of Veterinary Tropical Diseases, University of Pretoria, South Africa; 4 College of Veterinary Medicine and Biomedical Sciences, Colorado State University, United States

## Abstract

Salmonellosis is a common healthcare-associated infection in large-animal hospitals, and surveillance for *Salmonella* is an integral part of comprehensive infection control programmes in populations at risk. The present study compares the effectiveness of two culture techniques for recovery of *Salmonella* from environmental samples obtained in a large-animal referral veterinary hospital during a *Salmonella* outbreak. Environmental samples were collected using household cleaning cloths that were incubated overnight in buffered peptone water (BPW). Aliquots of BPW were then processed using two different selective enrichment and culture techniques. In the first technique (TBG-RV-XLT4) samples were incubated at 43 °C in tetrathionate broth and then Rappaport-Vassiliadis broth before plating on XLT4 agar. The second technique (SEL-XLD) involved incubation at 37 °C in selenite broth before plating on XLD agar. *Salmonella* was recovered from 49.7% (73/147) of samples using the TBG-RV-XLT4 technique, but only 10.2% (15/147) of samples using the SEL-XLD method. Fourteen samples (9.5%) were culture-positive using both methods, and 73 (49.7%) were culture-negative using both techniques. There were discordant results for 60 samples, including 59 that were only culture-positive using the TBG-RV-XLT4 method, and one sample that was only culture-positive using the SEL-XLD method. *Salmonella* was much more likely to be recovered using the TBG-RV-XLT4 method, and there appeared to be five times more false-negative results using the SEL-XLD technique. Environmental contamination with *Salmonella* may be underestimated by certain culture techniques, which may impair efforts to control spread in veterinary hospitals.

## Introduction

*Salmonella* is a common cause of nosocomial infection in large-animal hospitals and may result in hospital closure or restricted admissions (Benedict, Morley & Van Metre 2008; Dallap Schaer, Aceto & Rankin 2010; Steneroden *et al.* 2010; Tillotson *et al.* 1997). A number of large-animal hospitals routinely culture patient faeces and the hospital environment for *Salmonella* in order to detect nosocomial infection at the earliest stage (Dallap Schaer *et al.* 2010; Steneroden *et al.* 2010). This targeted surveillance to detect environmental contamination has been shown to be an important tool in detecting and mitigating outbreaks of *Salmonella* in veterinary hospitals (Dallap Schaer *et al.* 2010; Steneroden *et al.* 2010; Tillotson *et al.* 1997).

Routine, non-targeted sampling and culture of environmental samples collected from healthcare environments is not recommended by the Centers for Disease Control and Prevention (CDC) and other authorities (Sehulster *et al*. n.d.), but targeted microbiological sampling (e.g. methods used specifically to detect *Salmonella* contamination in order to detect or help mitigate nosocomial outbreaks) is considered a useful aid to infection control processes (Dallap Schaer *et al.* 2010; Steneroden *et al.* 2010; Tillotson *et al.* 1997) as long as there is a predefined protocol for sample collection and culturing and the analysis and interpretation of results are used as a basis for determining whether there is a need for intervention and which specific actions should be taken (Sehulster *et al*. n.d.).

A number of factors can affect surveillance results, including sample site selection, sampling frequency, sampling technique, sample storage and culture technique (Corrente *et al.* 2004; Ewart *et al.* 2001; Harvey & Price 1983; Ruiz *et al.* 1996). Isolation of *Salmonella* from faeces and the environment requires use of a selective enrichment medium and subsequent plating on selective agar. There are many options for media and culture techniques and methods for isolation of *Salmonella* from faeces and environmental samples, since they are not standardised (Davies *et al.* 2000; Waltman & Mallinson 1995). These methods vary greatly amongst laboratories and little information has been published about direct comparisons of techniques (Davies *et al.* 2000; Love & Rostagno 2008; World Organisation for Animal Health 2010; Rostagno *et al.* 2005; Singer *et al.* 2009; Voogt *et al.* 2002).

Inadequate *Salmonella* culture techniques that produce false-negative results may delay implementation of strategies to control nosocomial disease and so facilitate spread of *Salmonella* within the hospital and between patients (Dallap Schaer *et al.* 2010). The purpose of this study was to compare the recovery of *Salmonella* from environmental samples collected during a *Salmonella* outbreak in an equine hospital using two different culture methods.

## Materials and methods

### Study overview

In 2012 during an outbreak of healthcare-associated *Salmonella* infections at a large-animal referral hospital there was an opportunity to directly compare different culture methods for recovery of *Salmonella* from environmental samples. Environmental samples were collected from the animal care areas of the hospital and cultured using two different culture methods. One method was adapted from that previously described for environmental surveillance in a veterinary teaching hospital (Burgess, Morley & Hyatt 2004), and the other was the existing method being used in the diagnostic laboratory associated with the referral hospital. Samples were collected using commercially available household cleaning cloths, which were incubated overnight in buffered peptone water (BPW). Samples of BPW were then cultured by two different methods. In the first technique (TBG-RV-XLT4) samples were incubated at 43 °C in tetrathionate broth with brilliant green (TBG) and then Rappaport-Vassiliadis broth (RV) before plating on xylose-lysine-tergitol (XLT4) agar. The second technique (SEL-XLD) involved incubation in selenite broth (SEL) at 37 °C before plating on xylose-lysine-deoxycholate (XLD) agar.

### Sample site determination

Environmental samples were collected from 147 sites in the Onderstepoort Veterinary Academic Hospital. Sample sites were selected prior to sampling, considering hospital layout, traffic patterns for patients and personnel and how spaces are used in care for animals. These sites included areas used by animals only (e.g. stables), areas used by people only (e.g. offices) and areas used by both people and animals (e.g. treatment rooms). Within these sites hand-contact areas (e.g. telephones, keyboards, door handles) and foot-contact areas (e.g. floors) were sampled separately. High-risk areas such as high-traffic areas and those housing patients with gastro-intestinal disease were selected for more extensive sampling than low-risk areas (e.g. low-traffic areas).

### Sample collection

Samples were collected with dry new household cleaning cloths (Supawipes^®^, Pick ‘n Pay, Johannesburg, South Africa [SA]) in a similar manner to that previously described for collection of environmental samples in large-animal hospitals (Burgess *et al.* 2004). A single cloth was used at each site. Cloths were attached to a modified commercial floor mop to facilitate collection of floor and wall samples. This mop was disinfected with 70% ethanol between sampling of each site. Hand-contact areas were sampled with a cloth held in a gloved hand. After sampling each cloth was placed in a labelled sterile plastic bag (Whirl-Pak, Nasco, Fort Atkinson, WI, United States of America [USA]). Personnel changed gloves between each sample collection. An effort was made to sample approximately 80% of the surface area of each sample site.

### Culture methods

BPW (Oxoid Ltd., Basingstoke, Hampshire, United Kingdom [UK]) (90 mL) was added to each labelled plastic bag containing a cloth. These samples were incubated for 24 hrs at 37 °C and samples were then vortexed and aliquots were cultured using two different methods in parallel (TBG-RV-XLT4 method and SEL-XLD method). For the TBG-RV-XLT4 method, 100 uL of iodine iodide solution was added to 9 mL TBG (Selecta Media, Randburg, Gauteng, SA). The iodine iodide solution was prepared by mixing 6 g iodine (Merck, Modderfontein, Gauteng, SA) and 5 g potassium iodide (Merck, Modderfontein, Gauteng, SA) in 20 mL sterile distilled water. One mL of BPW was then added to the TBG and incubated for 24 hrs at 43 °C. The TBG was then vortexed and 0.1 mL was transferred to 10 mL of RV (Oxoid Ltd, Basingstoke, Hampshire, UK) and incubated for 24 hrs at 43 °C before it was vortexed and streaked for isolation on XLT4 agar (Selecta Media, Randburg, Gauteng, SA) and incubated overnight at 43 **°**C. Suspect colonies (pink colonies with or without black centres) were streaked for isolation onto Columbia blood agar plates (Selecta Media, Randburg, Gauteng, SA) and XLT4 agar and incubated for 24 hrs at 37 °C (Columbia blood agar) and 43 °C (XLT4 agar). For the SEL-XLD method, 1 mL of BPW was transferred to 9 mL of SEL (Oxoid Ltd, Basingstoke, Hampshire, UK) and incubated overnight at 37 °C before it was vortexed, streaked for isolation onto XLD agar (Oxoid Ltd, Basingstoke, Hampshire, UK) and incubated overnight at 37 °C. Suspect colonies (pink colonies with or without black centres) were streaked for isolation onto Columbia blood agar plates and XLD agar and incubated for 24 hrs at 37 °C.

For both methods identification of *Salmonella enterica* isolates was confirmed by biochemical testing using a commercial kit (API10S, BioMirieux, Marcy I’Etoile, Rhône-Alpes, France). If samples cultured positive using both methods, the isolate from the TBG-RV-XLT4 method was stored at -70 °C in brain-heart infusion broth (Oxoid Ltd, Basingstoke, Hampshire, UK) pending serotyping. Positive isolates from the SEL-XLD method were only stored and serotyped if the sample did not culture-positive using the TBG-RV-XLT4 method.

### Control samples

Positive and negative control samples were created by culturing American Type Culture Collection (ATCC) reference strains of *Salmonella* *Typhimurium* (ATCC 13311) (Teddington, Middlesex, UK) and *Escherichia coli* (ATCC 25922) respectively on blood agar. These cultures were sampled using cotton-tipped swabs (The Scientific Group, Vorna Valley, Gauteng, SA). The tips of the swabs were then cut-off, placed in sterile plastic bags containing BPW and processed using the same methods as for the environmental samples. Laboratory personnel were not blinded as to the identity of the control samples.

### Data management and analysis

Results of both culture methods were entered in a computer spreadsheet. Descriptive statistics were calculated, and differences in recovery by the different techniques were compared using McNemar’s test and Cohen’s kappa.

## Results

Positive and negative control samples were culture-positive and culture-negative respectively for *S. enterica* using both culture techniques. A total of 50.3% (74/147) of the environmental samples were culture-positive for *S*. *enterica*, with the TBG-RV-XLT4 method detecting *S. enterica* in 49.7% (73/147) of samples, and SEL-XLD detecting *S. enterica* in 10.2% (15/147) of samples. Overall 9.5% (14/147) of samples were culture-positive on both techniques, 49.7% (73/147) of samples were culture-negative using both methods, 40.1% (59/147) were only culture-positive using the TBG-RV-XLT4 method, and the one remaining sample (0.7%, 1/147) was only culture-positive using the SEL-XLD method. This marked discordance was found to be statistically significant using the McNemar test (*P* < 0.001), and the kappa statistic was 0.18.

Six different serotypes were identified amongst the isolates recovered in this study: *S.* *Heidelberg*, *S. Kibusi, S. Kottbus*, *S. Orion*, *S. Typhimurium* and *S. Virchow* (Table 1). The serotype of three isolates could not be determined by the reference laboratory using typing antisera. The majority (81.1%, 60/74) of *S. enterica* isolates obtained from culture-positive samples were serotype *S*. *Kibusi*.

**TABLE 1:** Serotypes of *Salmonella enterica* subsp. *enterica* obtained.

Serotype	All positive samples	Culture-positive samples stored from TET-RV-XLT4	Culture-positive samples stored from SEL-XLD
Heidelberg	1	1	0
Kibusi	60	60	0
Kottbus	3	3	0
Orion	4	3	1
Typhimurium	2	2	0
Virchow	1	1	0
Not determined	3	3	0
Total	74	73	1

## Discussion

This study showed that there was a marked difference in the ability to recover *Salmonella* from environmental samples, dependent on the culture method used. The TBG-RV-XLT4 culture method was more sensitive than the SEL-XLD culture method, with the latter method resulting in a large number of false-negative results. In a survey of laboratories in the USA regarding methods used for *Salmonella* culture from poultry samples, various formulations of selenite broth were used in > 1/3 of laboratories when culturing environmental samples (Waltman & Mallinson 1995). Veterinarians should therefore inquire about the methods being used at diagnostic laboratories for *Salmonella* culture, and this information should be used when selecting which laboratories to use.

False-negative results from environmental cultures can have important adverse effects on hospital infection control programmes. Firstly, if the environmental contamination is associated with healthcare-associated infections in patients, false-negative results for environmental cultures can contribute to the impression that salmonellosis is community-acquired rather than hospital-acquired, and so delay identification and mitigation of a nosocomial outbreak (Dallap Schaer *et al.* 2010). Secondly, false-negative environmental results may result in reopening of facilities that are still contaminated, with subsequent recurrence of nosocomial infection (Schott *et al.* 2001; Tillotson *et al.* 1997), which could necessitate repeat facility closure. Lastly, false-negative results delay the identification and hence cleaning and disinfection of contaminated areas (Dallap Schaer *et al.* 2010).

Early identification of contaminated stables in hospitals aids in resolving contamination. However, use of additional culture steps also has consequences, as this means it takes longer to obtain negative results. For example, even though the TBG-RV-XLT4 method had much greater sensitivity in this study when compared with the SEL-XLD method, results were obtained 24 hrs sooner using the SEL method. Delays in obtaining culture results can create important logistical problems in busy hospitals where patient turn-over is high. However, the magnitude of the difference in sensitivity outweighs the difference in culture times when considering the safety of patients and personnel.

A previous report demonstrated a difference in *Salmonella* recovery between two environmental sampling and culture systems in a large-animal hospital (Ruple-Czerniak *et al.* 2014). However, it was not possible to determine from that study the relative contributions of the sampling technique and the culture method to the overall detected difference in *Salmonella* recovery. This study demonstrates a clear difference in *Salmonella* recovery that can be attributed to culture methods.

A number of studies have investigated the use of different media for isolation of *Salmonella* from pigs, humans, chickens, reptiles and food products (Corrente *et al.* 2004; Davies *et al.* 2000; Love & Rostagno 2008; Oboegbulem 1993; Rostagno *et al.* 2005; Ruiz *et al.* 1996; Voogt *et al.* 2002). Comparisons of selective and enrichment media for isolation of *Salmonella* from horses are very limited. In a comparison of tetrathionate (TET) enrichment, RV selective incubation and SEL selective incubation of horse faeces, TET was found to be superior to RV for detection of *Salmonella,* and no *Salmonella* was isolated from samples selectively incubated in SEL (Babu *et al.* 2008).

This study suggests similar enhanced *Salmonella* recovery from an equine hospital environment using TET enrichment and RV selective incubation when compared with SEL selective incubation. The majority of *Salmonella* isolates obtained by environmental sampling were *S. Kibusi* (81%, 60/74). Different *Salmonella* serotypes vary in their tolerance for selective media and it is possible that the differences between the two techniques may not have been the same for other serotypes (Singer *et al.* 2009). However, TET broths have been shown to yield a much higher test sensitivity for isolation of *Salmonella* than SEL broths for a large number of serotypes (Carlson & Snoeyenbos 1974). In addition, it is possible that different serotypes were identified by the two different culture methods, but as positive isolates using the SEL-XLD method were only stored and serotyped if they were negative on the TBG-RV-XLT4 method it was not possible to determine this.

This study was carried out in a veterinary hospital during a nosocomial outbreak of salmonellosis and illustrated that the extent of environmental contamination would have been underestimated using the SEL-XLD method. This may have resulted in a less aggressive approach to the outbreak and so facilitated further spread of the organism and subsequent nosocomial infection. Hospital contamination was extensive at the time of this study; nevertheless a large difference was identified between culture methods. It is possible that the differences between the two methods may have been even further exacerbated if there were only low levels of environmental contamination. For example, it has been shown that large bacterial counts of *S. enterica* subsp. *arizonae* and lower numbers of competitors result in reduced ability to detect differences in efficacy of selective media (Snoeyenbos & Carlson 1972).

There were a number of differences between the two culture methods. It was not possible to determine the relative contributions of the different components of each culture method to the final observed difference in culture results. Further investigation would be required to determine if this difference was caused (all or in part) by the use of TBG enrichment versus SEL selective incubation, by use of two versus one selective enrichment steps, or by incubation at 43 °C versus 37 °C.

## Conclusion

In summary, this study identified an important difference in S*almonella* recovery from hospital environmental samples dependent on culture method. Use of a more sensitive culture technique should facilitate earlier recognition and mitigation of environmental *Salmonella* contamination, which may ultimately prevent the need for hospital closure.
